# Frontiers in Gastrointestinal Oncology: Advances in Multi-Disciplinary Patient Care

**DOI:** 10.3390/biomedicines6020064

**Published:** 2018-06-01

**Authors:** Nelson S. Yee, Eugene J. Lengerich, Kathryn H. Schmitz, Jennifer L. Maranki, Niraj J. Gusani, Leila Tchelebi, Heath B. Mackley, Karen L. Krok, Maria J. Baker, Claire de Boer, Julian D. Yee

**Affiliations:** 1 Division of Hematology-Oncology, Department of Medicine, Penn State Health Milton S. Hershey Medical Center, Experimental Therapeutics Program, Penn State Cancer Institute, The Pennsylvania State University College of Medicine, Hershey, PA 17033, USA; 2 Population Sciences Program, Penn State Cancer Institute, Department of Public Health Sciences, The Pennsylvania State University College of Medicine, Hershey, PA 17033, USA; elengeri@phs.psu.edu (E.J.L.); kschmitz@phs.psu.edu (K.H.S.); 3 Division of Gastroenterology and Hepatology, Department of Medicine, Penn State Health Milton S. Hershey Medical Center, The Pennsylvania State University College of Medicine, Hershey, PA 17033, USA; jmaranki@pennstatehealth.psu.edu (J.L.M.); kkrok@pennstatehealth.psu.edu (K.L.K.); 4 Division of General Surgery and Surgical Oncology, Department of Surgery, Penn State Health Milton S. Hershey Medical Center, The Pennsylvania State University College of Medicine, Hershey, PA 17033, USA; ngusani@pennstatehealth.psu.edu; 5 Department of Radiology, Penn State Health Milton S. Hershey Medical Center, The Pennsylvania State University College of Medicine, Hershey, PA 17033, USA; ltchelebi@pennstatehealth.psu.edu; 6 Department of Radiology, Medicine, and Pediatrics, Penn State Health Milton S. Hershey Medical Center, The Pennsylvania State University College of Medicine, Hershey, PA 17033, USA; hmackley@pennstatehealth.psu.edu; 7 Department of Medicine, Penn State Health Milton S. Hershey Medical Center, Penn State Cancer Institute, The Pennsylvania State University College of Medicine, Hershey, PA 17033, USA; mbaker@pennstatehealth.psu.edu; 8 Center Stage Arts in Health, Penn State Health Milton S. Hershey Medical Center, Department of Humanities, The Pennsylvania State University College of Medicine, Hershey, PA 17033, USA; cdeboer@pennstatehealth.psu.edu; 9 College of Liberal Arts, The Pennsylvania State University, State College, PA 16801, USA; jdy133@psu.edu

**Keywords:** gastrointestinal oncology, pancreatic carcinoma, hepatocellular carcinoma, biliary tract carcinoma, gastric carcinoma, colorectal carcinoma, stereotactic body radiation therapy, liver transplant, targeted therapy, psychosocial support

## Abstract

Cancers of the digestive system remain highly lethal; therefore, the care of patients with malignant diseases of the digestive tract requires the expertise of providers from multiple health disciplines. Progress has been made to advance the understanding of epidemiology and genetics, diagnostic and screening evaluation, treatment modalities, and supportive care for patients with gastrointestinal cancers. At the Multi-Disciplinary Patient Care in Gastrointestinal Oncology conference at the Hershey Country Club in Hershey, Pennsylvania on 29 September 2017, the faculty members of the Penn State Health Milton S. Hershey Medical Center presented a variety of topics that focused on this oncological specialty. In this continuing medical education-certified conference, updates on the population sciences including health disparities and resistance training were presented. Progress made in various diagnostic evaluation and screening procedures was outlined. New developments in therapeutic modalities in surgical, radiation, and medical oncology were discussed. Cancer genetic testing and counseling and the supportive roles of music and arts in health and cancer were demonstrated. In summary, this disease-focused medical conference highlighted the new frontiers in gastrointestinal oncology, and showcase the multi-disciplinary care provided at the Penn State Cancer Institute.

## 1. Introduction

The Multi-Disciplinary Patient Care in Gastrointestinal Oncology conference was held on September 29, 2017 at the Hershey Country Club in Hershey, Pennsylvania, U.S. This conference’s target audience included primary care physicians, gastroenterologists, medical oncologists, surgical oncologists, radiation oncologists, nurse practitioners, physician assistants, and nurses. The faculty members of the Penn State Cancer Institute and Penn State Health Milton S. Hershey Medical Center presented a variety of topics that focused on the frontiers in caring for patients with various gastrointestinal cancers. The purpose of this conference was to provide updates on new developments and emerging trends in caring for patients with various malignant diseases of the digestive system. The objectives of this program were to (1) recognize the risk factors and genetic mutations of cancers in the digestive system for prevention and early detection, (2) discuss diagnostic modalities and multi-disciplinary treatment of patients with digestive organ cancers, and (3) explore supportive interventions for patients with malignant diseases of the digestive system.

## 2. Population Sciences in Cancers of the Digestive System

### 2.1. Epidemiology of Cancers in the Digestive System

In 2018, the estimated number of new cases of cancer of the digestive system was the highest among all cancer sites in the United States [1]. Among the cancers of the digestive organs, colon cancer was the most prevalent. From 2007 to 2013, the five-year relative survival rates by all tumor stages at diagnosis for pancreatic cancer was the lowest among all cancer sites. The next lowest five-year relative survival rate was attributed to cancers in the liver, intrahepatic bile duct, esophagus, stomach, and lung. Lengerich, V.M.D., M.S., Associate Director of Health Disparities and Engagement, provided an overview of the epidemiology of digestive system cancer in the United States.

#### 2.1.1. Health Disparities in Appalachia

Lengerich presented public health data on various cancers of the digestive system in Appalachia, in which certain counties of central Pennsylvania are located. Compared with the general population in the United States, the Appalachian residents tend to have less contact with physicians, lower levels of preventive care, and less health insurance coverage for non-elderly people. Lengerich reported epidemiological data on health disparities in the Appalachian communities. In particular, the incidence and mortality rates of colon cancer and rectal cancer in the Appalachia were greater than of the U.S. population [2,3,4]. These data suggest relatively little use of screening interventions for colorectal cancer in rural Appalachia. This may be related to the low level of awareness of regular screening for colorectal cancer among the general population in Appalachia. Other contributing factors may include low availability of screening centers, long distances to health care facilities, high rates of unemployment and poverty, and inability to afford travel to screening facilities [5]. 

Various strategies attempted to reduce health disparities in cancer in the Appalachian communities were presented. An active area of investigation is the use of screening interventions for prevention and early detection of colorectal cancer, including a national, multimedia campaign called Screen for Life, which aims to educate people aged 50 or older about the importance of regular screening tests for colorectal cancer [6]. The effectiveness and methods for dissemination of Screen for Life materials in rural Appalachia were examined by a network of investigators working in medically under-served regions. These reports indicated a substantial potential for the Screen for Life materials and campaign, though limited at the local level, in rural Appalachia [7,8,9]. However, these observations led to the hypothesis that the number of individuals in rural Appalachia seeking colorectal cancer screening could be increased by disseminating Screen for Life materials at state, regional, and community levels through health care practices and organizations [5]. Additionally, primary care physicians may help engage patients in screening for colorectal cancer by encouraging the use of fecal occult blood testing when colonoscopy is not possible and systems-based reminders that provide electronic resources that are not visit-dependent [10].

Limited access to healthcare services is challenging for those who live in rural communities, and strategies to involve Appalachian populations as participants in research and overcome that disparity were discussed. These include community-based participatory research in rural communities with the goals of increasing awareness of community assets and enhancing treatment-related care and psychosocial care [11]. Through collaboration with community physicians, these initiatives may help improve patients’ access to tertiary care and clinical studies at academic cancer centers, and facilitate education of patients and the general population. Other strategies include raising funding support for research on health disparities, increasing availability of screening interventions for early detection of cancer, and development of community plans to enhance survivorship by improving the long-term health and well-being of cancer survivors in rural locations.

#### 2.1.2. Key Points and Recommendations

Despite advances in screening, early detection, diagnosis, and treatment of various malignant diseases, digestive system cancer incidence and mortality rates remain among the highest. The disparity in cancer care for people in Appalachia is a longstanding problem, which is partly a socioeconomic issue. Malignant diseases in the digestive organs besides colorectal cancer are largely unexplored in the Appalachian population. Special emphasis of research efforts, judicial allocation of funding support for research, and prudent distribution of resources will hopefully make a meaningful impact on health by lessening the burden of cancers of the digestive system. 

### 2.2. Exercise in Cancer Patients and Survivors

Growing evidence suggests the roles of exercise in improving treatment response and reducing treatment-related toxicities in cancer patients, as well as preventing disease recurrence in cancer survivors. The report of a recent survey demonstrates that oncologists have little knowledge regarding exercise counseling, and they are not routinely discussing exercise with their patients [12]. Schmitz, Associate Director of Population Sciences, described the benefits of exercise in patients diagnosed with various malignant diseases with an emphasis on colon cancer.

#### 2.2.1. Clinical Studies of Exercise in Cancer Patients and Survivors

Schmitz provided the existing and emerging evidence for the safety and efficacy of exercise training during and following systemic treatment of cancer. A large number of studies demonstrated that exercise is safe in patients with breast cancer, including those who had exercise training during chemotherapy or radiation therapy, as well as those who had exercise training following completion of chemotherapy or radiation therapy [13]. The adverse events reported in those studies, such as plantar fasciitis and other musculoskeletal injuries, were mild and rare. Notably, for women who have had surgical resection of their axillary lymph nodes and/or radiation therapy to the axilla, aerobic, and/or resistance training did not cause or worsen lymphedema. Additionally, studies of exercise interventions in survivors of prostate cancer showed that exercise is safe in this population [13]. Importantly, a significant association between high levels of exercise and low risks of cancer-specific mortality and cancer recurrence were observed in patients with breast cancer or prostate cancer as well as other malignant diseases [14].

Colon cancer is the third most common cancer, which is associated with a fairly good prognosis. Yet, few clinical studies have evaluated the potential benefits of exercise for reducing chemotherapy-related toxicities and improving treatment efficacy. None of the trials addressed safety or adverse events except one report, which indicated that there was no significant abnormality in electrocardiograms during maximal aerobic fitness testing [15]. However, one study reported a statistically significant association between high exercise levels and a low risk of recurrence and all-cause mortality of colorectal cancer [16]. A clinical study to investigate the effects of aerobic exercise on tumor recurrence and as the molecular and cellular pathways associated with physical activity among patients with stage II and III colon cancer was completed (www.clinicaltrials.gov NCT02250053). Analysis of the results of this important study pends.

#### 2.2.2. Clinical Studies of Exercise in Cancer Patients at Penn State Cancer Institute

Currently, a clinical study is ongoing at the Penn State Cancer Institute and regional facilities to further investigate the safety and efficacy of resistance training in patients receiving chemotherapy for treatment of colorectal cancer (www.clinicaltrials.gov NCT03291951). This is a randomized, open-label, controlled trial of resistance training intervention in patients with newly-diagnosed stage II or III colon cancer receiving chemotherapy. The primary goal of this study is to examine the effects of resistance training on chemotherapy-related outcomes, including dose delays, dose reductions, early stoppage, and grade 3 and 4 toxicities. This clinical trial consists of two aspects: an in-person and telephone-based intervention to promote home-based resistance training, and a wait-list, control group. In the resistance training group, the subjects will work with an exercise professional on the same day as a chemotherapy infusion session, and the subjects will complete a series of exercises at home twice weekly throughout the intervention. For the control group, the subjects will be told to continue whatever exercise program they have been undertaking up to enrolling in the study, but to not increase exercise or begin weight-lifting over the period of study participation.

Another study is currently open for enrollment at Penn State Cancer Institute for patients who receive chemotherapy for treatment of any solid tumor including cancers in the digestive system (www.clinicaltrials.gov NCT03461471). The primary objective of this study is to assess the safety, feasibility, and acceptability of an exercise program within the course of chemotherapy. This is an open-label, single group study for patients diagnosed with a solid tumor malignancy at stages I to IV. Feasibility will be accomplished if one-third of the patients receiving chemotherapy actually perform the prescribed exercise (one exercise session per week for four weeks). Other outcome measures include changes in pain, physical function, nausea, emesis, and arthralgia as well as alterations of chemotherapy (dose delays and changes).

#### 2.2.3. Key Points and Recommendations

Numerous clinical studies have demonstrated the benefits of exercise training in terms of physical functions and quality of life for patients with cancer. Significant association has been reported between high exercise levels and reduced risks of cancer recurrence and cancer-specific mortality. The evidence to date supports the recommendation of regular exercise for people with cancer as well as those who have completed cancer treatment. Ongoing studies are being designed and conducted to test the hypothesis that exercise is safe and improves treatment response, quality of life, and survival in patients, and reduces toxicities for various malignant diseases including those in the digestive system.

## 3. Diagnostic Evaluation of Esophageal, Pancreatic, Biliary Tract, and Hepatocellular Carcinoma

Technological advances have been made in the diagnostic evaluation of cancers of the digestive system. Improved accuracy of diagnosis and staging of malignant diseases in the upper gastrointestinal organs has been enabled by endoscopy along with various imaging modalities. Surveillance of hepatocellular carcinoma (HCC) and liver transplantation have become increasingly important for early detection and treatment of this disease.

### 3.1. Diagnostic Evaluation and Staging for Cancers of Esophagus, Pancreas, and Biliary System

Endoscopy plays a central role in the diagnosis of cancer in both the upper and lower gastrointestinal tracts. Maranki, Medical Director of Endoscopy, discussed the diagnostic evaluation and staging for cancers of the esophagus, pancreas, and biliary system. In particular, endoscopic ultrasonography (EUS) is an important diagnostic modality for patients with esophageal and pancreatic carcinoma by evaluating the extent of tumor invasion and any involvement of the regional lymph nodes, and by enabling tumor biopsy through fine needle aspiration. The use of a new technique via SpyGlass^TM^ cholangioscopy has improved the diagnostic yield of biliary tract carcinoma through high-resolution direct visualization with biopsy of the bile ducts.

#### 3.1.1. Diagnosis and Staging of Esophageal Cancer

Esophageal carcinoma, either squamous cell carcinoma or adenocarcinoma, is typically diagnosed by esophagogastroduodenoscopy with tissue biopsy. Computed tomography (CT) of the chest and abdomen and positron emission tomography (PET) in combination with CT scans are indicated to evaluate any metastatic disease. EUS plays an important role in staging the disease based on depth of invasion of the esophageal wall and involvement of any regional lymph nodes if there is no evidence of metastatic disease. Endoscopic mucosal resection of early stage tumors (T1a, T1b) can be therapeutic and curative.

#### 3.1.2. Diagnosis and Staging of Pancreatic Cancer

Imaging studies for diagnosis of pancreatic carcinoma include dual-phase helical CT scans, transabdominal ultrasonography (US), EUS-guided fine needle aspiration (FNA), endoscopic retrograde cholangiopancreatography (ERCP), magnetic resonance cholangiopancreatography (MRCP), and PET scans. The accuracy of these imaging studies for diagnosis of pancreatic carcinoma (PC) was compared [17]. In particular, the sensitivity and specificity of EUS-guided FNA are 92% and 100%, respectively; those of ERCP are 70% and 94%, respectively. Moreover, the performance of EUS for pancreatic adenocarcinoma was compared to CT and MRI scans with regard to nodal staging, vascular invasion, and resectability.

As shown in Table 1, EUS appears less sensitive but more specific than CT scans for staging of lymph node involvement, detecting vascular invasion, and determining resectability of tumors. For nodal staging, EUS is more sensitive and less specific than MRI scans; for detecting vascular invasion, EUS is less sensitive and more specific than MRI scans. Notably, the performance of EUS is dependent on the operator, and EUS should be considered complementary to either CT or MRI scans for the staging of pancreatic adenocarcinoma.

#### 3.1.3. Diagnosis and Staging of Cholangiocarcinoma

If a lesion in the biliary tract is identified on imaging studies, such as US, CT, MRI, or MRCP, EUS-guided FNA or ERCP with biliary brushings are indicated for establishing the diagnosis. CT-guided biopsy of the tissue is considered if necessary. All lesions suspicious of cholangiocarcinoma should be further evaluated with MRI or MRCP. However, diagnosis of cholangiocarcinoma by tissue biopsy can be challenging, and tissue may be obtained via several techniques. Of note, EUS-guided FNA is not advisable for hilar and intrahepatic lesions if the tumor is considered resectable. This is due to concerns of tumor seeding the needle tract. A new technique via SpyGlass^TM^ cholangioscopy has been shown to improve diagnostic yield for cholangiocarcinoma.

SpyGlass^TM^ cholangioscopy uses fiberoptic technology for high-resolution direct visualization of the bile ducts. Since the launch of the Spyglass^TM^ Direct Visualization System, the sensitivity for detecting cholangiocarcinoma has been improved [18,19]. ERCP with Spyglass^TM^ Direct Visualization System enables the direct visualization of the bile ducts, thus facilitating tissue biopsy and therapeutic intervention. With the implementation of a new digital system, the SpyGlass^TM^ cholangioscopy provides a sensitivity and specificity of 90% and 95.8%, respectively, for diagnosis of malignant disease in the bile ducts [20].

#### 3.1.4. Key Points and Recommendations

For diagnostic and staging evaluation of esophageal carcinoma, PET and CT scans and EUS are the standard of care. Besides CT scans, the role of EUS is particularly valuable for evaluating patients with localized pancreatic carcinoma that appears resectable on the initial imaging study. A new technique using ERCP with Spyglass^TM^ Direct Visualization System has improved the sensitivity and specificity for the diagnosis of cholangiocarcinoma.

### 3.2. Hepatocellular Carcinoma

HCC is a major cause of cancer-related mortality worldwide. The incidence, disease burden, and mortality of HCC have been rising in the United States. Krok, Medical Director of Liver Transplantation, provided a hepatologist’s perspective on HCC. Krok’s presentation focused on the risk factors of HCC, screening modalities of HCC, and liver transplant for treatment of selected patients.

#### 3.2.1. Risk Factors for HCC

An update on the risk factors for HCC in the United States was provided. For persons above 65 years old, diabetes and obesity represent the major risk factors for HCC, and they are followed by hepatitis C virus (HCV), alcoholism, smoking, hepatitis B virus (HBV), and rare genetic disorders [21]. In obese men, liver cancer is associated with the highest relative risk of death among all types of cancer [22]. For patients with hepatitis C viral (HCV) infection, HCC is generally developed in the setting of advanced hepatic cirrhosis [23]. HBV DNA is considered a key risk for the development of HCC, and the baseline serum level of HBV DNA is correlated with the incidence of HCC over time.

#### 3.2.2. Screening of HCC

Surveillance of HCC has been shown to improve outcomes by early tumor detection, reduction of total mortality, and improved survival [24]. Surveillance guidelines for high-risk patients by ultrasonography (US), with or without serum alpha-fetoprotein (AFP) or prothrombin induced by vitamin K absence-II (PIVKA-II), have been recommended by various cancer organizations in the United States, Europe, Japan, and other Asian-Pacific countries. These screening tests are indicated at various time intervals, from every 3 months or 6 months to 12 months. Besides the U.S., other imaging modalities for surveillance of HCC, including CT and MRI scans, were described. Their advantages and disadvantages were compared, with MRI scans showing the highest sensitivity of detecting HCC [25]. Special emphasis was placed on contrast-enhanced ultrasonography (CEUS), which demonstrates higher sensitivity, negative predictive value, and overall accuracy that standard US [26]. Furthermore, a meta-analysis indicated that CEUS and gadoxetate-enhanced MRI scans show the highest sensitivity and positive predictive value for detecting HCC [27].

#### 3.2.3. Liver Transplantation for Treatment of HCC

Liver transplant is a viable treatment option for many patients with HCC. Liver transplant can be a curative intervention of choice for HCC, especially for patients with cirrhosis that cannot easily have a surgical resection. The five-year survival rate is about 75%, which is comparable to patients without HCC who undergo liver transplant. Both cadaverous and living donor liver transplant can be offered. In the United States, there have been more than 1,000 liver transplants per year for patients with HCC since 2008. A total of 15,045 liver transplants have been performed for patients with HCC. Currently, there are 14,104 patients waiting for a liver transplant with a diagnosis of HCC. Currently, the Milan criteria are the most commonly used for evaluating candidates for liver transplant [28]. Once a patient is considered to be within the Milan criteria and meets all criteria for listing for liver transplant, they will be listed for transplant. Various modalities, including transcatheter arterial chemoembolization (TACE), radiofrequency ablation, and Yttrium-90, may be used for bridge treatment prior to liver transplantation. For HCC with vascular invasion, tumor recurrence following liver transplant progressively increases with time [29].

#### 3.2.4. Key Points and Recommendations

The major risk factors for HCC in the United States include obesity, hepatitis C viral infection, and alcoholism. Interventions and research efforts should be focused on developing strategies, especially behavioral modification for preventing HCC. Imaging studies using US, CT, and MRI scans along with serum tumor markers, including AFP and PIVKA-II, facilitate early detection of HCC. For individuals at risk of developing HCC, active surveillance is indicated with the hope of detecting small tumors at a localized stage, so that they can be cured by liver transplantation or surgical resection. Whereas liver transplantation is a curative intervention for both HCC and the underlying hepatic cirrhosis, organ donation remains a limiting factor and requires continued public support.

## 4. Therapeutic Interventions by Surgical Resection, Radiation Therapy, and Systemic Treatment

A number of advances have been made in the localized, systemic, and targeted treatment of cancers of the digestive organs. New developments in surgical management as well as pre-operative and post-operative interventions have been applied for improved clinical outcomes. Refinement of criteria for radiation therapy continues, and new technologies for precise radiotherapy while minimizing toxicity has been investigated. Constant new developments have occurred in systemic therapies for improving the treatment response for patients with advanced or metastatic diseases.

### 4.1. Surgical Gastrointestinal Oncology

Surgical interventions have been playing a crucial role in the treatment of various malignant diseases in the digestive system. Gusani, Group Leader of Liver, Pancreas, and Foregut Program and Surgical Oncologist, presented new paradigms in the surgical management of gastrointestinal cancer. Gusani discussed the importance of treatment of the whole patient involving prehabilitation and survivorship, and treatment of advanced tumors by considering the whole range of treatment options. These include multi-visceral and extended resections and the use of neoadjuvant therapy. Improved surgical outcomes through better understanding of anatomy, surgical techniques, and peri-operative medicine, and minimally invasive surgery were also discussed.

Gusani presented a surgeon’s perspective on various aspects of gastrointestinal oncology. The surgical techniques for resection of gastric and gastroesophageal junction adenocarcinoma, hepatocellular carcinoma, metastatic colon adenocarcinoma in liver, and pancreatic tumors (adenocarcinoma, cysts, and neuroendocrine tumor) were described. Highlights of their presentation include the role and goal of physical activity in survivorship of patients who have undergone surgical resection of gastrointestinal carcinoma, and minimally invasive surgery that offers benefits in peri-operative outcomes while preserving oncologic outcomes.

### 4.2. Radiation Gastrointestinal Oncology

Technological advances have been made and applied in radiation oncology, and increasing evidence has indicated the clinical efficacy of stereotactic body radiation therapy (SBRT) in cancers of the digestive system. Mackley, Attending and Consultant Radiation Oncologist, provided an update on the general indications of radiation therapy for upper gastrointestinal malignancies based on the National Cancer Consortium Network (NCCN) treatment guidelines. This included current recommendations that support SBRT as an option in the treatment of unresectable PC and HCC. Tchelebi, Attending and Consultant Radiation Oncologist, further discussed the technical delivery of SBRT and the clinical evidence that supports this emerging treatment modality.

#### 4.2.1. SBRT in Pancreatic Carcinoma

SBRT involves very high dose radiation delivered to a highly conformal treatment volume over a short treatment course (about one to five fractions over one to two weeks). As compared to three-dimensional conformal radiation therapy, SBRT may achieve superior tumor control by delivering a higher biological effective dose over a shorter overall treatment time with increased sparing of adjacent of critical organs. Moreover, Tchelebi presented data from recent clinical studies about using SBRT in the neoadjuvant setting for patients with borderline resectable pancreatic carcinoma (BRPC) and unresectable locally advanced pancreatic carcinoma (LAPC) [30]. Clinical data from an earlier study and recent studies on the use of SBRT as definitive treatment of locally advanced unresectable pancreatic carcinoma were also presented. Tchelebi concluded that SBRT improves resectability of BRPC and enables 5–10% of patients with unresectable LAPC to undergo resection. Additionally, clinical trials are ongoing to investigate chemotherapy (modified FOLFIRINOX) with or without SBRT in LAPC (www.clinicaltrials.gov NCT01926197), and using adjuvant SBRT for patients following radical resection of pancreatic carcinoma with advanced tumor stages or lymph node involvement (www.clinicaltrials.gov NCT02461836).

#### 4.2.2. SBRT in Hepatocellular Carcinoma

External beam radiation therapy (EBRT) has been used for treatment of patients with HCC in various settings. EBRT may be considered for definitive treatment of HCC unsuitable for resection, liver transplant, or radiofrequency ablation; as a bridge to liver transplant; as definitive therapy for HCC unsuitable/refractory to TACE; or when there is tumor invasion of the portal vein. EBRT may be used at low doses for symptomatic HCC. Tchelebi reviewed the data from multiple clinical studies that investigated SBRT for HCC [30]. They discussed the advantages of SBRT for HCC including the effectiveness for large tumors (>10 cm), HCC with thrombosis in the portal vein, or when other local therapeutic modalities are contraindicated or less effective; sparing of adjacent un-involved liver; and producing complete pathological responses in patients who undergo liver transplant. Tchelebi concluded that SBRT can be used in HCC when other local therapies are not feasible (such as for large tumors or thrombosis in the portal vein), or in conjunction with TACE, or as a bridge to liver transplant.

### 4.3. Medical Gastrointestinal Oncology

Systemic therapies play important roles in various gastrointestinal malignancies at different tumor stages. Progressive advances have been made in improving the therapeutic efficacy of systemic treatment using chemotherapy, targeted therapy, and immunotherapy [31,32,33,34,35,36,37,38,39,40,41]. Results of recent clinical studies suggested new treatment options for patients with various malignant disease in the digestive system. Yee, Team Leader of Gastrointestinal Oncology and Attending and Consultant Medical Oncologist, provided an overview of the standard treatment of pancreatic, gastroesophageal, hepatobiliary, and colorectal carcinoma. Yee presented and discussed the most recent evidence from international medical conferences and medical literature on chemotherapy, targeted therapy, and immunotherapy in gastrointestinal oncology.

#### 4.3.1. Systemic Treatment of Pancreatic Carcinoma

Systemic chemotherapy is an essential component of standard treatment for patients with PC diagnosed at all stages. New developments have been focusing on targeted agents directed against the tumor microenvironment and cancer stem cells. In a phase II clinical study of 246 patients, PEGylated hyaluronidase (PEGPH20), that degrades hyaluronan in the tumor-associated stroma, in combination with *nab*-paclitaxel and gemcitabine, significantly prolonged progression-free survival (PFS) and overall survival (OS), as compared to *nab*-paclitaxel and gemcitabine [42]. Moreover, this study suggested that hyaluronic acid is a potential predictive biomarker of tumor response to PEGPH20.

Results of a first-in-human clinical trial using napabucasin that targets cancer stem cells by inhibiting the activation of the signaling molecule STAT3 have been reported. In a phase 1b/II study of 66 patients with metastatic PC, the STAT3 inhibitor napabucasin that targets cancer stem cells, in combination with *nab*-paclitaxel and gemcitabine, produced anti-tumor response, with an overall response rate of 55%, disease control rate 93%, and progression-free survival 7.1 months [43]. A phase III study to investigate napabucasin in combination with nab-paclitaxel and gemcitabine versus nab-paclitaxel and gemcitabine is currently recruiting worldwide including at the Penn State Cancer Institute (www.clinicaltrials.gov NCT02993731).

#### 4.3.2. Systemic Treatment of Gastric and Gastroesophageal Carcinoma

For localized gastric carcinoma (GC) and gastroesophageal junction carcinoma (GEJC), peri-operative systemic chemotherapy using a combination regimen consisting of cisplatin and 5-fluorouracil is the standard of care. In a phase III study of 716 patients, a group of patients received peri-operative FLOT (docetaxel, oxaliplatin, 5-fluorouracil), and another group received peri-operative ECF (epirubicin, cisplatin, 5-fluorouracil) or ECX (epirubicin, cisplatin, capecitabine) [44]. Peri-operative FLOT improved clinical outcomes with a significant prolongation of progression-free and overall survival, reduction in the progression of disease during or following pre-operative chemotherapy, and an increase in pT0, pT1, and R0 resection. Results of this study support peri-operative FLOT as the new standard of systemic treatment for patients with resectable GC or GEJC.

The anti-PD-1 antibodies pembrolizumab significantly improved the response rate and overall survival beyond second line treatment of patients with advanced GC or GEJC expressing PD-L1. Results of this study led to approval of pembrolizumab by the Food and Drug Administration (FDA) in this patient population [45].

#### 4.3.3. Systemic Treatment of Hepatocellular and Biliary Tract Carcinoma

The anti-PD-1 antibody nivolumab was investigated in a study of 262 sorafenib naïve or sorafenib-treated patients with advanced HCC. Nivolumab produced tumor responses regardless of etiology of HCC or tumor expression of PD-L1 [46]. Results of this study led to FDA approval of nivolumab for treatment of patients with HCC following prior sorafenib regardless of PD-L1 status.

In a phase III trial, the clinical efficacy of adjuvant capecitabine was evaluated in resected biliary tract carcinoma (BTC). In this study, 447 patients were randomized to receive adjuvant capecitabine versus observation. Intrahepatic (19%), hilar (28%), extra-hepatic (35%), gallbladder (18%). Capecitabine 1250 mg/m^2^ on day 1 through day 14 of every 21-day cycle for a total of 8 cycles. The results of this study indicated that adjuvant capecitabine significantly prolonged overall survival in resected biliary tract carcinoma [47].

#### 4.3.4. Systemic Treatment of Colorectal Carcinoma

Nivolumab, an anti-PD-1 antibody, has been recently FDA-approved for treatment of patients with metastatic colorectal carcinoma (CRC) with microsatellite instability-high (MSI-H) or mismatch repair-deficiency (dMMR) that has progressed following fluoropyrimidime, oxaliplatin, and irinotecan. This indication is based on the data from a multicenter, open-label, single-arm, phase 2 clinical trial (CheckMate 142) [48].

The benefits of three months vs. six months of adjuvant chemotherapy using FOLFOX (5-fluorouracil, leucovorin, and oxaliplatin) or CAPOX (capecitabine and oxaliplatin) in stage III or high-risk stage II CRC were investigated [49,50]. Results of these studies indicated that three months of adjuvant chemotherapy is not inferior to six months of treatment. As a result, three months of adjuvant chemotherapy is recommended for low-risk disease (T1-3, N1). However, the use of three months of adjuvant chemotherapy for high-risk disease (T4 or N2 tumors) should be tailored to the individual patient.

### 4.4. Key Points and Recommendations

Surgical interventions continue to play crucial roles in the treatment of various malignant diseases in the digestive system. Besides the importance of surgical techniques for resection, treatment of the whole patient involving prehabilitation, survivorship, and physical activity, as well as minimally invasive surgery, offer benefits in both peri-operative and oncological outcomes. While three-dimensional conformal radiation remains the conventional therapeutic modality in gastrointestinal oncology, SBRT that involves a highly conformal treatment volume over a short treatment course has emerged as a treatment option that provides benefits in efficacy and safety, particularly in PC and HCC. Results of the recent clinical trials that investigated chemotherapy, targeted therapy, and immunotherapy have led to U.S. FDA approval of nivolumab in HCC and CRC with dMMR or MSI-H, and pembrolizumab in GC or GEJC expressing PD-L1, as well as solid tumors that display dMMR. Peri-operative FLOT for resectable GC and GEJC, adjuvant capecitabine for resected (R0) BTC, and three months of adjuvant FOLFOX or CAPOX for low-risk CRC are expected to become the new standard of care.

## 5. Gastrointestinal Cancer Genetics

Convincing evidence indicated that the development of cancer typically involves genetic disposition and behavioral and environmental factors. Although certain risk factors are potentially modifiable [51], genetic testing of hereditary gastrointestinal cancer syndromes has become an integral part of patient care in oncology [52]. Technological advances in the next generation sequencing of human genomes have improved genetic testing for the screening of various malignant diseases, particularly cancers of the digestive system. Baker, Director of Cancer Genetics, provided an overview of the genetic syndromes that predispose a person to GC, CRC, and PC. They discussed the practical aspects and clinical application of next generation sequencing (NGS) panels for hereditary gastrointestinal cancer.

### 5.1. Cancer Genetic Testing and Counseling 

The genetics of gastric, colorectal, and pancreatic carcinoma were presented. For GC, approximately 3–5% of patients have a hereditary risk, and three heritable syndromes are known to predispose primarily to GC: hereditary diffuse gastric cancer (HDGC), familial intestinal gastric cancer (FIGC), and gastric adenocarcinoma and proximal polyposis of the stomach (GAPPS). Specific genes and types of mutations were identified in HDGC (*E-cadherin* and *α-E-catenin* genes), and GAPPS (promoter 1B mutations the *APC* gene), whereas no known genetic variants have been identified in FIGC. The diagnostic criteria for both FIGC, and GAPPS were described, as was the testing criteria for HDGC [53]. Other cancer syndromes and the associated genes that predispose a person to GC were also presented. These include familial adenomatous polyposis (*APC*), hereditary breast and ovarian cancer syndrome (*BRCA1*, *BRCA2*), juvenile polyposis syndrome (*SMAD4*, *BMPR1A*), Li-Fraumeni syndrome (*TP53*), Lynch syndrome (*MLH1*, *MSH2*, *MSH6*, *PMS2*, *EPCAM*), and Peutz-Jeghers syndrome (*STK11*).

In addition to GC, the genetic syndromes associated with hereditary CRC [52] and PC [52,54] were presented. Other hereditary cancer syndromes with gastrointestinal involvement [55] and the standards for informed consent for genetic testing in gastrointestinal practice [52] were also described.

### 5.2. Next-Generation Sequencing Panels

The traditional approach to cancer genetic testing that entails analysis of one gene per condition at a time using Sanger sequencing was compared to the contemporary approach using NGS panels. Given the increased number of genes analyzed with NGS panels, the process of informed consent, out of necessity, had to change dramatically. No longer was each gene discussed independently, but rather genes were categorized into groups of high, moderate, or low susceptibility regarding their impact on cancer risk. Risks, benefits, and limitations of NGS panels were also discussed, using case examples for illustration. Potential benefits include the ability to simultaneously analyze multiple cancer susceptibility genes in a more cost-efficient manner, thus increasing the likelihood of identifying one or more cancer predisposition syndromes within a family. Potential risks and limitations, though, include the higher likelihood of identifying one or more variants of uncertain significance and the likelihood that a mutation may be identified in a gene for which our knowledge base is still evolving with regards to the spectrum of associated cancers, the estimated lifetime risks of these cancers, as well as appropriate management guidelines. In addition, unexpected findings may be identified such as a gene mutation that is not consistent with the family history of cancer or a mutation in a recessive gene that has reproductive implications.

Lastly, choosing a laboratory for NGS panels is a practical aspect of cancer genetic testing and a number of questions will need to be considered and addressed [56]. Evaluation of the technology being used involves consideration of the testing platform, depth of coverage, and presence of a deletion/duplication assay. Analysis of the genes involves considering the number of genes examined, whether a cancer site-specific panel or a pan-cancer panel would be more appropriate, looking at the proportion of genes that are “medically actionable”, and whether the option exists to modify the panel or create a custom panel. Other pertinent factors for consideration include the cost of testing and insurance coverage, the turn-around time, the rate of variants of uncertain significance (VUS), the reliability of the laboratory, and the ease of laboratory use.

### 5.3. Key Points and Recommendations

The genetic syndromes and the associated genetic mutations for GC, CRC, and PC have been identified. Clinicians should be vigilant about individuals with cancer and their family history of cancer, and should be prompt to refer patients to cancer geneticists for genetic testing and counseling. Patients with germline mutations that predispose to various malignant diseases will likely benefit from screening tests for early detection of tumors and taking appropriate measures for preventive and therapeutic interventions. Cancer genetic testing using NGS panels is expected to produce potential benefits by concurrent analysis of multiple cancer susceptibility genes in a cost-efficient manner, thus increasing the likelihood of identifying cancer predisposition syndromes within a family.

## 6. Supportive Care in Oncology

Complementary to the treatment of cancer, emotional, psychological, and social support are essential for the well-being of patients, particularly those with gastrointestinal cancers. In clinical practice, patients with advanced cancer are recommended to receive palliative care early in the disease course and concurrent with anti-cancer treatment [57]. At the Penn State Cancer Institute, supportive care services are provided by healthcare providers from multiple disciplines to patients with malignant diseases. They include palliative care physicians and nurses, clinical psychologists, kinesiologists, nutritionists, physical therapists, artists and music therapists, acupuncturists, and population scientists for smoking cessation. A support group that focuses on gastrointestinal cancer patients and survivors is underway at the Penn State Cancer Institute. The goals include improving the quality of life for cancer patients and survivors as well as the well-being of their caretakers.

### 6.1. Music and Arts in Health and Oncology

A program that integrates music, arts, and creative writing has been established at the Milton S. Hershey Medical Center through the Center for Humanistic Medicine and Center Stage Arts in Health. The goal of this program is to provide supportive care by psychological and cognitive improvement. This program is particularly needed for patients with gastrointestinal malignancies that are associated with high levels of psychosocial distress, disease burden, and mortality rate. de Boer, Director of Center Stage Arts in Health, provided an overview of the music and arts programs at Penn State Health including the Cancer Institute.

### 6.2. Center Stage Arts in Health

Center Stage Arts in Health is a multi-faceted program that aims to nourish well-being through the arts. At Center Stage, professional musicians play cheerful and reflective music with a variety of genres and instruments in the lobby and numerous clinical and family areas. The staff of Center Stage visit patients upon their admission to the hospital, and patients have the opportunity to choose artwork created by regional artists to hang in their room during their hospital stay [58]. Murals are created in the clinical and family waiting areas (https://sites.psu.edu/centerstage/murals/), and original art is commissioned for display throughout the hospital. A summer lunchtime concert series is conducted in the outdoor courtyard, featuring local professional ensembles including jazz, classical, and soft rock music. At Center Stage and the Penn State Cancer Institute, a set of arts workshops of multiple modalities, including an expressive workshop program, is offered to cancer patients and their caregivers. A brief video introduction of the Center Stage program can be watched at https://www.youtube.com/watch?time_continue=8&v=HT3wJn8OoF4.

Faculty-led investigations are ongoing to observe, explore, and quantify the impact of the arts on the experience of patients and caregivers. During the summer of 2017, a Pennsylvania State University undergraduate student, Julian Yee, who majors in psychology, participated in research as a Center Stage Intern. During the internship, Yee assisted the Center Stage Team to promote music and arts in health. First of all, Yee observed the pertinence of the social interactions between musical artists and visitors, staff, and patients. From there, Yee recorded the data of musical acts against the audience’s reactions. Then, they transported art supplies to guide cancer survivors and their families to make art crafts on Survivorship Day. Finally, they helped paint a mural in a hallway so when children pass by, they are reminded that the hospital can be a colorful environment.

### 6.3. Key Points and Recommendations

Emotional, psychological, and social support is an essential component of the multi-disciplinary care of patients, and particularly those with cancers in the digestive system. For patients with advanced gastrointestinal cancer, palliative care is recommended concurrent with anti-cancer treatment early in the disease course. The Center Stage Arts in Health integrates music, arts, and creative writing with the aim to nurture the well-being of patients through psychological and cognitive improvement.

## 7. Conclusions

Cancers of the digestive system continue to represent a major cause of physical and psychosocial burden. Healthcare practitioners and scientists with expertise in multiple disciplines play critical roles in providing optimal care for patients with these malignant diseases. Recent advances have been made in gastrointestinal cancer epidemiology and genetics, diagnostic evaluation, treatment modalities, and supportive care. This conference paper summarizes the presentations by the faculty members of the Penn State Health Milton S. Hershey Medical Center with a focus on gastrointestinal oncology. These specialists provided updates on new developments in (1) health disparities and resistance training, (2) diagnostic evaluation and screening procedures, (3) conventional and novel therapeutic modalities, (4) cancer genetic testing and counseling, and (5) music and arts in health and cancer. In summary, this medical conference highlighted the new frontiers in the multi-disciplinary care for patients with gastrointestinal cancers.

## Figures and Tables

**Table 1:** Comparison of endoscopic ultrasonography (EUS) and either computed tomography (CT) or magnetic resonance imaging (MRI) scans for pancreatic adenocarcinoma.

	EUS vs. CT		EUS vs. MRI	
	Sensitivity	Specificity	Sensitivity	Specificity
**Nodal staging**	24% vs. 58%	88% vs. 85%	36% vs. 15%	87% vs. 97%
**Vascular invasion**	58% vs. 86%	95% vs. 93%	42% vs. 59%	97% vs. 84%
**Resectability**	87% vs. 90%	89% vs. 69%	NA	NA

NA: not available. This table is modified from reference [17].
